# Production of Bio-Based Pigments from Food Processing Industry By-Products (Apple, Pomegranate, Black Carrot, Red Beet Pulps) Using *Aspergillus carbonarius*

**DOI:** 10.3390/jof6040240

**Published:** 2020-10-22

**Authors:** Ezgi Bezirhan Arikan, Oltan Canli, Yanis Caro, Laurent Dufossé, Nadir Dizge

**Affiliations:** 1 Department of Environmental Engineering, Mersin University, Mersin 33343, Turkey; ndizge@mersin.edu.tr; 2 Environment and Clean Production Institute, The Scientific and Technological Research Council of Turkey, Marmara Research Center, Kocaeli 41470, Turkey; oltan.canli@tubitak.gov.tr; 3 Département Hygiène Sécurité Environnement (HSE), IUT La Réunion, Université de La Réunion, 40 avenue de Soweto, BP 373, F-97455 Saint-Pierre, Réunion, France; yanis.caro@univ-reunion.fr; 4 Laboratoire de Chimie et de Biotechnologie des Produits Naturels, Chemistry and Biotechnology of Natural Products (CHEMBIOPRO), Université de La Réunion, ESIROI Agroalimentaire, 15 Avenue René Cassin, CS 92003, F-97744 Saint-Denis, Réunion, France

**Keywords:** *Aspergillus carbonarius*, bioconversion, food processing industry by-product valorization, filamentous fungi, bio-based pigment

## Abstract

Food processing industry by-products (apple, pomegranate, black carrot, and red beet pulps) were evaluated as raw materials in pigment production by the filamentous fungi *Aspergillus carbonarius.* The effect of fermentation conditions (solid and submerged-state), incubation period (3, 6, 9, 12, and 15 d), initial substrate pH (4.5, 5.5, 6.5, 7.5, and 8.5), and pulp particle size (<1.4, 1.4–2.0, 2–4, and >4 mm) on fungal pigment production were tested to optimize the conditions. Pigment extraction analysis carried out under solid-state fermentation conditions showed that the maximum pigment production was determined as 9.21 ± 0.59 absorbance unit at the corresponding wavelength per gram (AU/g) dry fermented mass (dfm) for pomegranate pulp (PP) by *A. carbonarius* for 5 d. Moreover, the highest pigment production was obtained as 61.84 ± 2.16 AU/g dfm as yellowish brown at initial pH 6.5 with < 1.4 mm of substrate particle size for 15-d incubation period. GC×GC-TOFMS results indicate that melanin could be one of the main products as a pigment. SEM images showed that melanin could localize on the conidia of *A. carbonarius*.

## 1. Introduction

Color has always been the basis for the evaluation of both aesthetics and quality for humanity [1]. Pigments that can be defined as colorant compounds are used in many industries such as textiles, cosmetics, dyes, pharmaceuticals, food etc. [2,3]. Before the discovery of synthetic colorants in the mid-19th century, pigments were obtained from natural sources such as animals, plants, and rocks [4]. The discovery of a synthetic pigment named mauveine in 1856 [5] triggered the industries’ usage of synthetic pigments [6]. However, recent studies have shown that some synthetic pigments may have carcinogenic, teratogenic, and allergenic effects [2,7]. For this reason, utilization of some synthetic pigments in food, pharmaceutical, and cosmetic products is limited or prohibited by organizations such as the World Health Organization (WHO), the Food and Agriculture Organization of the United Nations (FAO), and the US Food and Drug Administration (FDA) [8,9]. The increasing legal requirements and consumer awareness in recent years have encouraged industries to use a larger amount of natural pigments. Hence, recent studies have focused on production of cost-effective natural pigments [10,11,12,13], also called bio-based pigments.

Natural pigments that can be obtained from plants, animals, and microorganisms are mostly biocompatible, biodegradable, environmental-friendly, and they have low toxicity [11,14,15]. The usage of plants and animals for natural pigment production has many disadvantages such as the non-stability and high solubility of pigments, dependency on the season, and the loss of certain species for large scale production [16]. However, microbial pigment production is considered more advantageous due to their higher growth rate, the fact that they are unaffected by the seasonal changes, and the high stability of produced pigments [3,7]. A literature survey showed that among the microorganisms, fungi are mostly preferred for industrial-scale production of natural pigments because algae requires sunlight and bacteria are more vulnerable to environmental conditions.

On the one hand, it is estimated that the market of natural pigments presents the highest growth rate of around 7% per year [17]. On the other hand, the production of natural pigments at industrial-scale has depended on the design of a cost-effective production process [18,19,20]. It is known that the cost of microbial bio-pigment production has been affected by 38–73% of raw material selection [7]. Thus, the raw materials selected as a substrate in the production of bio-pigments using fungi should be both inexpensive and rich in carbon and nitrogen sources. In this context, agricultural or food by-products originated from industries have recently gained great attention due to their applicability for obtaining new valuable products with a zero-waste strategy [21]. Therefore, food by-products were chosen as a low-cost substrate for pigment production in this study.

Filamentous fungi are known to be producers of many types of bio-pigments, such as carotenoids, melanins, flavins, phenazines, quinones, monacins, and indigo [22,23]. Hence, filamentous fungi such as *Monascus*, *Aspergillus*, *Penicillium*, *Neurospora*, *Eurotium*, *Drechslera*, and *Trichoderma* have been found to be the subject of many studies as a potential producer of bio-pigments [19,24,25,26,27,28]. Among the filamentous fungi, *Aspergillus* is the fungal genus most commonly found on foods [29] and they are able to produce various pigments that contain hydroxyanthraquinoid [22]. Recently, many studies have focused on potential pigment production from *Aspergillus spp.* and the optimizing of production [28,30,31,32]. However, to the best of our knowledge, pigment production from food by-products as substrates using *Aspergillus carbonarius* filamentous fungus has not yet been studied.

Based on the aspects cited above, the main objective of this study is to evaluate the potential ability of pigment production by the filamentous fungi *Aspergillus carbonarius*, using food processing industry by-products including apple, pomegranate, black carrot, and red beet pulp. Furthermore, the effect of fermentation conditions, incubation time, initial pH, and pulp particle size to optimize pigment production were evaluated.

## 2. Materials and Methods

### 2.1. Fungal Species and Inoculum

Filamentous fungi *Aspergillus carbonarius* M333 were obtained from the Department of Environmental Engineering Laboratory, Mersin University, Mersin, Turkey. This fungus was maintained on a potato dextrose agar (PDA, Merck, Darmstadt, Germany) slant at 4 °C and sub-cultured monthly. *A. carbonarius* was transferred to PDA medium in Petri dishes in a UV laminar chamber (Faster, UCS2–4, Ferrara, Italy) and incubated at 25 ± 1 °C for 7 d. After incubation, spores of fungus were individually harvested from the surface of the Petri dishes. A suspension of spores was prepared in sterile distilled water containing Tween 80 (0.1%, Fisher). The concentration of fungus was then adjusted to 1 × 10^6^ colony-forming unit/mL by using a Thoma cell counting chamber [33]. The spore suspension of *A. carbonarius* was used for inoculation for further studies.

### 2.2. Substrate

In this study, apple, pomegranate, black carrot, and red beet pulp were selected as substrates for pigment production by fungi. Pulps were kindly provided by Anadolu Etap Agriculture and Fruit Products Industry and Trade Inc. in Mersin City, Turkey. To evaluate the effect of the initial carbon to nitrogen (C:N) ratio, elemental analysis of each type of pulp was performed at this stage. For further studies, pulps were dried at 60 ± 1 °C for 24 h in an oven (Figure S1), followed by particle size reduction using a kitchen blender and sieved (Tyler mesh 10–12) [34] to obtain particles of 1.4 mm diameter. All dried pulp particles were stored at 4 °C prior to use.

### 2.3. Selection of Pulp Type

The experimental flow chart during this study is illustrated in Figure S2. In the first stage of the experiment, pigment production capacities of *A. carbonarius* on each pulp (apple, pomegranate, black carrot, and red beet) were evaluated by solid-state fermentation (Figure S2A) based on previous studies [35]. The particle size of all pulps was chosen to be under 1.4 mm in order to increase the penetration of fungal hypha. All types of pulp particles (5 g) were added in Erlenmeyer flasks (250 mL) individually and all flasks subsequently were autoclaved (Sanyo, MLS-3781L, Moriguchi, Japan) at 121 °C for 20 min. After cooling to room temperature, sterilized distilled water was added to each flask to adjust the initial moisture content of the substrate to 50% (*w/w*, on a dry basis) at aseptic conditions [36,37]. Then, spore suspension of fungus (1 mL) was added into the flasks. Some flasks were not inoculated, and these flasks were used as blanks. All flasks were incubated at 25 ± 1 °C in a static incubator (Sanyo, MIR152, Japan) for 5 d. After incubation, the complete solid mass (including fungal biomass and pulp mass) in each flask was harvested, dried at 60 ± 1 °C in an oven for 24 h, and used for pigment extraction and analysis (Figure S2A). Additionally, the harvested wet mass was used directly for pigment extraction in order to test extraction efficiency. For the rest of the study, the pulp particle type that has the highest pigment production was named as the optimum pulp. Further experiments were performed with the optimum pulp for evaluating the impact of fermentation strategy, incubation period, particle size of the substrate, and initial pH of the substrate on pigment production.

### 2.4. Effect of Fermentation Strategy and Incubation Period

After the optimum pulp type was determined, the effect of fermentation strategy (solid-state and submerged-state) and incubation period (3, 6, 9, 12, and 15 d) were investigated for pigment production capacities of fungus. For this purpose, 5 g of pulp (particle size < 1.4 mm) was transferred into Erlenmeyer flasks (250 mL). All flasks were sterilized at 121 °C for 20 min and cooled to room temperature. Solid-state fermentation (SSF) was carried out by adding sterilized distilled water to these flasks in order to obtain an initial moisture of 50%. For submerged-state fermentation (SmF), 50 mL of sterilized distilled water was added to flasks to reach the final working volume. Then, each flask was inoculated with 1 mL spore suspension of *A. carbonarius*. The flasks were incubated in a shaking incubator at 100 rpm and 25 ± 1 °C for SmF and in a static incubator at 25 ± 1 °C for SSF for 15 d. During the incubation period, the contents of flasks were harvested every 3 d and dried at 60  ±  1 °C for 24 h. Dried masses were utilized for pigment extraction and analysis (Figure S2B).

### 2.5. Effect of Substrate Particle Size

To determine the effect of particle size on pigment production, the optimum dried pulp type was sieved and classified as < 1.4 mm, 1.4–2.0 mm, 2–4 mm, and > 4 mm. Each particle size was evaluated for pigment production. Optimum pulp type at different particle sizes was weighed as 5 g and transferred to flasks. Following the sterilization at 121 °C for 20 min, sterile distilled water was added to the flasks, according to the optimum fermentation condition determined in the previous stage (solid-state or submerged-state). After inoculation of 1 mL of the spore suspension of *A. carbonarius*, all flasks were incubated at 25 ± 1 °C. During the incubation period, pigment extraction and estimation analysis were conducted to dried mass every 3 d (Figure S2C).

### 2.6. Effect of Initial Substrate pH

After determining the optimum pulp type, fermentation condition, incubation period, and pulp particle size for pigment production, the initial pH of the substrate was tested. For this purpose, 5 g of pulp particles and a known volume of distilled water according to the optimum fermentation condition were added to flasks. Then, the pH value of each flask was individually adjusted to 4.5 (original pH), 5.5, 6.5, 7.5, and 8.5 using a pH/Cond 340i Handheld Multimeter. The solution of sodium hydroxide (0.1 N) (Merck, Germany) and hydrogen chloride (0.1 N) (37%, Merck, Germany) was used for pH adjustment. After sterilization of all flasks that contain pulp particles at different initial pH levels, each flask was inoculated with spore suspension (1 mL) and incubated at 25 ± 1 °C for the optimum incubation period (Figure S2D). During the incubation period, the mass was separated from flasks, dried, and used for pigment extraction.

In the last stage of the experiment, pigment production was carried out at optimal conditions during the optimum incubation period (Figure S2E). The obtained pigment supernatant was used for GC×GC-TOFMS analysis, and dyeing tests. The obtained fungal biomass and pulp after fermentation were used for scanning electron microscopy (SEM) analysis and elemental analysis, respectively.

### 2.7. Instrumental Analysis

The morphological characterization of dried fermented mass obtained from optimal conditions was observed by SEM (Zeiss Supra 55, Oberkochen, Germany). Images were taken by applying an electron beam with an acceleration voltage of 5 kV. In addition, SEM analyses were performed on the unfermented optimum type of pulp and *A. carbonarius* growing in PDB to compare morphological changes.

The elemental analysis of each type of pulp was performed before fermentation due to evaluating C:N ratio utilization in the selection of pulp type experiments. Moreover, it was performed both before and after fermentation for the optimum pulp type, which provides higher pigment extraction. Elemental analysis was performed using a TruSpec Micro (LECO, St. Joseph, MI, USA) elementary analyzer (for C, H, N, and S weight percentages).

Two-dimensional gas chromatography (GC×GC) was used for the qualitative analysis of the extract. Approximately 10 mg of pulp, without any pre-treatment, was weighed and dissolved with 1.0 mL of hexane:acetone (1:1) solvent mixture. Liquid nitrogen used for cold pulses was automatically filled. The Agilent 7890 B (Palo Alto, CA, USA) Gas Chromatograph System was equipped with a LECO Pegasus^®^ BT 4D mass spectrometer (Leco, St. Joseph, MI, USA) dual-stage, quad jet thermal modulator and with a split/splitless injector. The GC primary column had 30 m × 0.25 mm id. × 0.25 μm film thickness Rxi^®^-17Sil MS (Restek Corp., Bellefonte, PA, USA). The GC secondary column had 0.75 m × 0.25 mm id. × 0.25 μm film thickness Rxi^®^-5Sil MS (Restek Corp, Bellefonte, PA, USA) mounted in a separate oven installed within the main GC oven. The carrier gas was helium and set 1 mL/min. Injection speed was 3 μL/s and the inlet purge time was 60 s. A 1 μL injection was made in splitless mode with an inlet temperature of 250 °C. The temperature program of the first column was as follows: 40 °C kept for 4 min, then raised at 8 °C/min up to 310 °C kept for 20 min. The temperature of the second oven was programmed with an offset of 10 °C and the modulator temperature offset was 25 °C relative to the first GC oven temperature. The second-dimension separation time (modulation time) was 5 s divided into a hot pulse time of 1.50 s and a cold pulse time between the stages of 1 s. The transfer line from the secondary oven into the mass spectrometer was maintained at 280 °C. The ion source was operated at 250 °C. The electron energy was −70 eV. The data acquisition rate was 200-scans/s, covering a mass range of 50–550 m/z.

### 2.8. Analytical Techniques

During the pulp type selection experiments, different pigment extraction protocols were performed to evaluate the effect of wet or dry fermented solids. During the following experiments, pigment extraction protocol was performed on a dry basis. Pigment extraction was performed according to the method reported by Kantifedaki et al. [34] with slight modifications. After the fermentation of every case, solid material was weighed as 0.5 g (on wet or dry basis), transferred into a tube and mixed with 5 mL of ethanol (95%). The mixture was placed in an ultrasonic bath at 60 Hz (Witeg, Wertheim, Germany) for 30 min at 25 °C. Then, the mixture was mixed on a rotary shaker at 180 rpm for 1 h at 30 °C followed by centrifugation at 6000 rpm for 20 min. After centrifugation, the supernatant was used for pigment estimation by measuring the absorbance with a UV–vis spectrophotometer (DR3900, Hach Co, CO, USA) along with utilizing a quartz cuvette. Yellow, orange, and red pigments were determined by measuring the absorbance in three different wavelengths, 400 nm, 475 nm, and 500 nm, respectively, taking into consideration the dilution factor of the sample [38]. In every step, the unfermented substrate was subjected to sterilization, but inoculation was not carried out. These unfermented pulp particles were subjected to pigment extraction. The results were expressed as absorbance unit at the corresponding wavelength per gram (AU/g). In this study, intracellular pigment production was assessed due to the application of pigment extraction to only fermented solids including fungal biomass and pulp mass.

### 2.9. Dyeing Tests

The wool fabric sample obtained from a local firm was cut into a size of 10 cm × 10 cm (3 g) and it was immersed into a glass beaker containing 50 mL extracted dye solution (10 mg/L). Then, this glass beaker that contained a dye solution liquor volume to fabric weight ratio of 50 mL/3 g was placed in a water bath and left at 93 °C for 1 h. For the uniform dyeing, the sample was stirred regularly. The dyed wool fabric piece was firstly washed with non-ionic detergent and then washed with tap water to remove the detergent [39]. Thereafter, the dyed fabric piece was dried at 60 °C for 24 h.

Furthermore, the dyeing test was also conducted with the mordanting process. For this purpose, the wool fabric was supplied by BOSSA Trade and Industry Enterprises Turkısh Inc. The fabrics (6 g) were mordanted at 50 °C for 60 min with iron sulfate (100 mL, 25 mg/L) [40]. After mordanting, the dyeing process of the mordanted fabric was carried out with an extracted dye solution (10 mg/L), so that the final dye solution liquor volume to fabric weight ratio was 100 mL/6 g. The dye bath was run for 60 min at 85 °C. After dyeing, color fastness to wash was assessed by obeying ISO 105-C10:2006 method.

## 3. Results and Discussion

### 3.1. Optimal Fungal Species and Pulp Type

Elemental analysis of pulps performed before fermentation are shown in Table 1. Results show that each type of pulp had essential nutrient content for fungal growth [41]. In addition to this, after the inoculation of *A. carbonarius* onto different pulps, it was observed that fungus was grown on each different pulp type and covered the surface of the pulps within 3 d. This observation (Figure S3) supports the results of elemental analysis.

**Figure 1:**
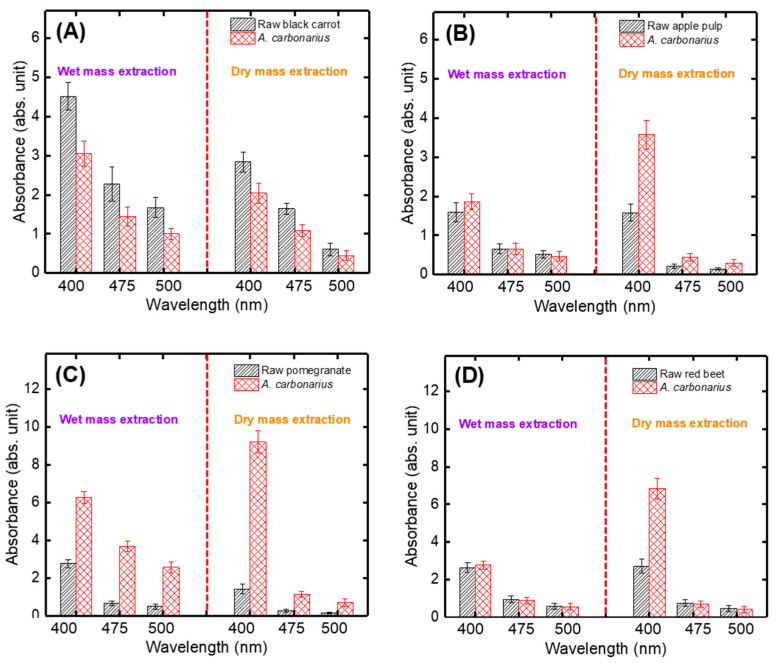
Evaluation of wet and dry mass pigment extraction methods during solid-state fermentation using *A. carbonarius* cultivated on (**A**) black carrot, (**B**) apple, (**C**) pomegranate, (**D**) red beet pulps (experimental conditions: incubation time, 5 d).

Figure 1 shows the results of pigment extraction obtained from wet and dry fermented mass after SSF using *A. carbonarius* cultivated on the pulp of black carrot (Figure 1A), apple (Figure 1B), pomegranate (Figure 1C), and red beet (Figure 1D). The literature describes that nitrogen and carbon sources have effects on secondary metabolite regulation [42]. Therefore, it is possible that different levels and types of pigments could be produced by the fungus grown on the different types of pulps, which have different contents of C and N sources.

Generally, a high C:N ratio has been reported to induce pigment production for filamentous fungi [43]. For example, Palacio-Barrera et al. [44] studied the effect of C:N ratio on pigment production by *Aspergillus chevalieri* and they found that a high C:N ratio (20/1, glucose/yeast extract) was optimum for inducing pigment production. However, it was not the case in this work for all type of pulps. The highest pigment production by *A. carbonarius* was on pomegranate, red beet and apple pulp, respectively. Nevertheless, the highest initial C:N ratio was red beet (20.63/1), pomegranate (16.87/1) and apple (11.53/1), respectively. This could be attributed to the decreasing pH levels during the fermentation of red beet pulps [45]. In this study, a more complex substrate such as a real industrial pulp was used for pigment production. It is known that pigment production is affected not only by the C:N ratio, but also by other factors such as types of C and N sources and the presence of organic acids and minerals [46]. For this reason, it is possible to obtain different results in pigment production from synthetic media and from more complex substrates.

On the other hand, the highest pigment extraction was mostly achieved with dry fermented mass compared to wet fermented mass, for apple (Figure 1B), pomegranate (Figure 1C), and red beet (Figure 1D) pulp. This can be explained as the wet mass (0.5 g on wet basis) used in the extraction method contained more water, whereas the dry mass (0.5 g on dry basis) contained less water due to drying. Hence, it can be concluded that the dry mass, as a result of dehydration, may contain more concentrated pigment due to the lack of water. Pigment extraction analysis carried out under SSF conditions showed that the maximum pigment production was determined as 9.21 ± 0.59 AU/g dry fermented mass (dfm) in pomegranate pulp by *A. carbonarius* for 5 d (Figure 1C) at 400 nm. It was also found that the highest absorbance and color were determined at 400 nm as yellow hue for all pulp types. It is known that yellow hydroxyanthraquinone (HAQ) pigments are produced by many species of *Aspergillus* [47]. Hence, the following studies were performed with pomegranate pulp (PP) for optimal pigment production.

### 3.2. Optimal Fermentation Strategy and Incubation Period

The cost and yield of bio-pigment production depend on the fermentation strategy used in production [35]. To date, different incubation periods have been tested for the production of pigment from different fungal species and wastes/pulps. For example, Babitha et al. [36] investigated pigment production by *Monascus purpureus* from jack fruit seed and they found that the maximum pigment production was on the sixth day. Gmoser et al. [48] found that the highest pigment (0.7 mg carotene/g waste) was produced by *Neurospora intermedia* from waste bread on the sixth day. In the study conducted by Padmavathi and Prabhudessai [49], the highest pigment production by *Monascus sanguineus* from potato peel was obtained on the 15th day.

**Figure 2:**
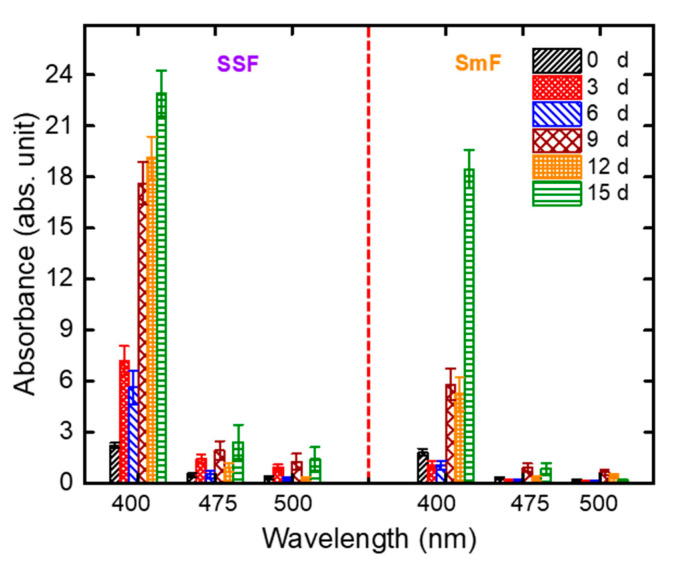
Evaluation of pigment production of *A. carbonarius* on pomegranate pulp (PP) at solid-state fermentation (SSF) and submerged-state fermentation (SmF) conditions (experimental conditions: pH: 4.5; shaking speed for SmF: 100 rpm).

Therefore, pigment production by *A. carbonarius* on PP was evaluated with SSF and SmF for 3, 6, 9, 12, and 15 d. In addition, the effect of incubation time on pigment production was limited to 15 d in this study due to the fact that a short fermentation time is desired in industrial production to obtain a competitive production [14]. Figure 2 illustrates the effect of two fermentation strategies (SSF and SmF) on pigment production over 15 d. SSF exhibited higher yellow pigment production (22.9 ± 1.34 AU/g dfm) than SmF. In addition, it was determined that pigment production mostly increased with increasing incubation time for both fermentation strategies.

Pigments are synthesized as secondary metabolites by the fungus [50] and these metabolites often produce at the stationary phase of fungal growth as a result of nutrient limitations and/or under stress conditions [48]. Furthermore, secondary metabolism is commonly associated with sporulation processes for microorganisms, including fungi [51]. It is known that pigment production can be related to the formation of both sexual and asexual spores for some fungal species [51]. On the other hand, *Aspergillus* species can produce asexual spores, which are called conidia [52], from their conidiophores as a result of differentiation of their aerial hyphae. For example, Teertstra et al. [53] showed melanin pigments being produced in the conidia of *Aspergillus niger*. For these reasons, two possible reasons can explain the increasing of pigment production when the incubation day increases: (i) for both fermentation strategies, the stationary phase began after 6 d (Figure 2) and pigment production increased with increasing incubation day because of decreasing nutrients or stress conditions, (ii) conidia that contain pigments were produced after 6 d. In addition, it is known that conidia are only formed in the air [48,54]. Due to the presence of water in SmF, conidia were not produced by *A. carbonarius* until day 12 (Figure 2). Therefore, SSF was found to be more successful in pigment production than SmF. Consequently, further studies were conducted with SSF for maximum pigment production.

### 3.3. Optimal Substrate Particle Size

**Figure 3:**
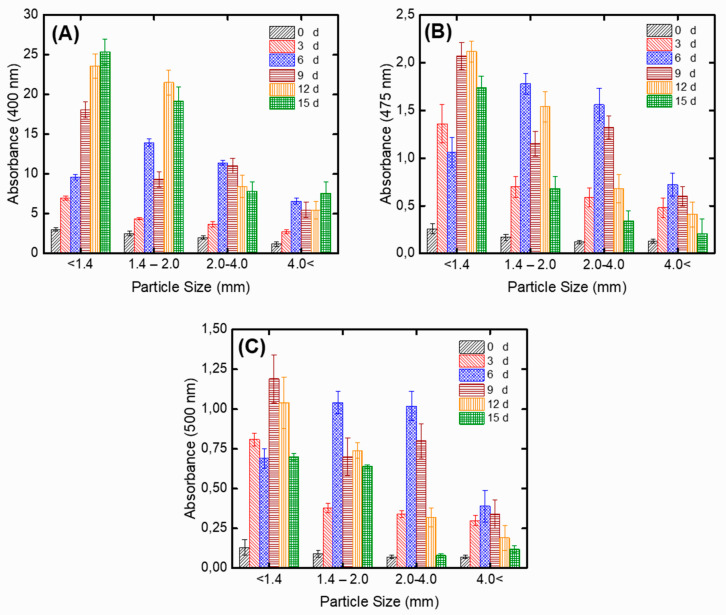
The effect of PP particle sizes on pigment production at (**A**) 400 nm, (**B**) 475 nm, (**C**) 500 nm during SSF with *A. carbonarius* (experimental conditions: pH: 4.5).

Figure 3 illustrates the effect of PP particle sizes (<1.4, 1.4–2, 2–4, and >4 mm) on pigment production during SSF with *A. carbonarius*. The smallest particle size (<1.4 mm) supplied higher pigment production, particularly in the case of the yellow pigment (25.38 ± 1.60 AU/g dfm for 15 d) (Figure 3A). Moreover, 2.12 ± 0.11 AU/g dfm (Figure 3B) and 1.19 ± 0.15 AU/g dfm (Figure 3C) were measured for orange and red pigments for 12 and 9 d, respectively. The same trend was observed by Kantifedaki et al. [34] and they reported that the smallest particle size (<2 mm) exhibited higher pigment synthesis (9 AU/g dfm for 16 d). This could be attributed to the fact that smaller substrate particles provided a larger surface area for the fungal attack to the substrate [55]. Due to obtaining higher pigment production with the smallest particle size, < 1.4 mm of PP was used for the production of pigments in the subsequent experiments.

### 3.4. Optimal Initial Substrate pH

**Figure 4:**
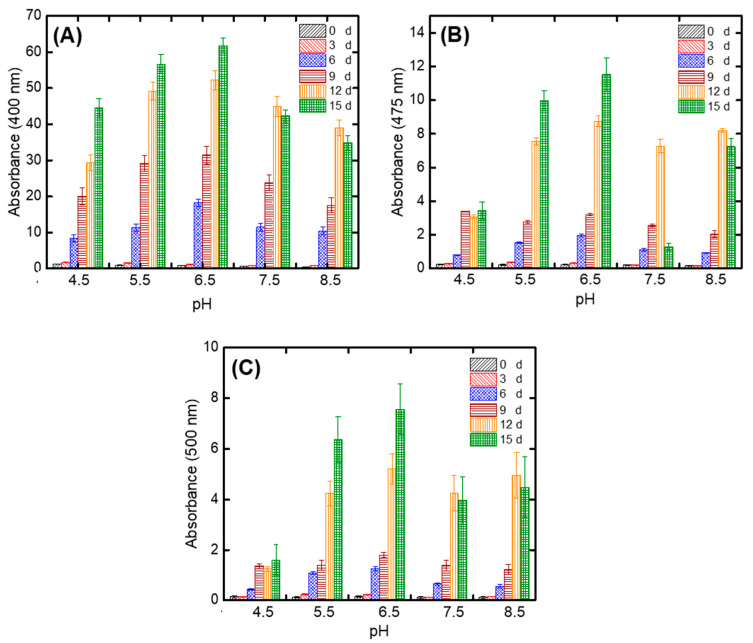
The effect of initial substrate pH on pigment production at (**A**) 400 nm, (**B**) 475 nm, (**C**) 500 nm during SSF with *A. carbonarius* (experimental conditions: particle size: < 1.4 mm).

The optimization of initial pH is an important parameter to increase pigment production. Figure 4 shows the effect of initial pH (4.5, 5.5, 6.5, 7.5, and 8.5) of PP on pigment production during SSF with *A. carbonarius*. During the incubation period, the highest pigment production was determined as 61.84 ± 2.16 AU/g dfm at 400 nm for 15 d for initial pH 6.5 (Figure 4A). Moreover, 11.52 ± 1.01 AU/g dfm (Figure 4B) and 7.56 ± 1.03 AU/g dfm (Figure 4C) were measured for orange and red pigments for 15 d, respectively. It was found that as pH increased between pH 4.5–6.5, pigment production increased. However, pigment production decreased after pH 6.5. Therefore, optimum pH was determined as pH 6.5 for the pigment production at the maximum yield in this study. A similar result was obtained, so that the production of pigment by *Aspergillus nidulans* increased when the initial pH of the substrate was at 6.8 compared to pH 8.0 [56]. Furthermore, Afshari et al. [57] studied pigment production from another filamentous fungal species *Penicillium aculeatum* and they found that the best production of yellow pigment was obtained with a pH value of 6.5. The effects of pH on fungal pigment production are connected with changes in enzyme activity [56]. It is known that the optimal pH range is 5.0–6.5 for most fungi [58]. Therefore, it is thought that the growth of fungi on PP and its enzyme activity for pigment production could affect pigment production.

### 3.5. C:N Ratio Utilization

Carbon and nitrogen are essential for fungal growth and pigment production. Elemental analyses of the unfermented and fermented pomegranate pulp are summarized in Table 2. The elemental analysis of the PP showed that their composition changed after fermentation. PP lost a percentage of nitrogen (from 3.02% to 1.60%), hydrogen (from 6.08% to 5.09%), sulfur (from 0.02 % to 0.00 %), and carbon (from 50.94% to 42.46%) after fermentation. It is known that the C:N ratio affects the biosynthesis of many metabolites, such as pigments, in fungi [59]. It was detected that the C:N ratio significantly increased from 16.87/1 to 26.54/1 after fermentation in this study. These results demonstrate that the stress condition for the pigment production in this experiment could be the nitrogen decrease [43].

### 3.6. Characterization of Fungal Biomass

**Figure 5:**
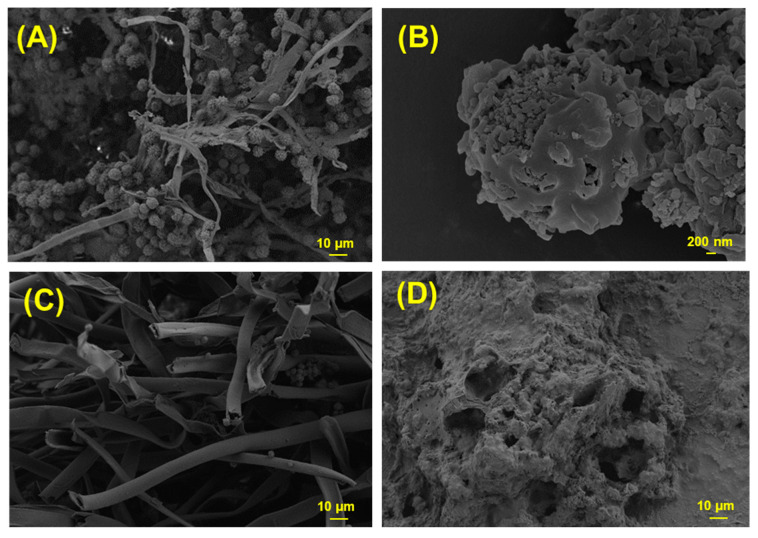
SEM images of (**A**) fermented *A. carbonarius* on PP under optimal conditions, (**B**) pigmented conidia of fermented *A. carbonarius* on PP under optimal conditions, (**C**) *A. carbonarius* grown on YMB, (**D**) unfermented PP.

To characterize fungal biomass, SEM is the one of most appropriate techniques. Figure 5 shows the SEM image of the fermented *A. carbonarius* on PP under optimal conditions. Fermented *A. carbonarius* on PP (Figure 5A) has more conidia whereas *A. carbonarius* grown on YMB has more hyphae (Figure 5C). This result supports our hypothesis that pigments were produced in conidia. Furthermore, Figure 5B shows the pigmented conidia of fermented *A. carbonarius* on PP. Unfermented PP (Figure 5D) shows a perforated structure.

It is known from the literature that *Aspergillus* species can produce melanin [30]. However, Pihet et al. [60] studied melanin production in *Aspergillus fumigatus* and SEM analysis results show that conidia that do not have smooth walls contain melanin. Therefore, this could support our second hypothesis that *Aspergillus carbonarius* could produce melanin from pomegranate pulp.

GC×GC-TOFMS is a reliable method for the detection of organic compounds in complex matrices [61], and its results are given in Table 3 for the extracted solution from fermented *A. carbonarius* on PP under optimal conditions. It is known that pomegranate contains sugars such as xylose, arabinose, and glucose [62]. GC×GC-TOFMS results show that fermentation was conducted successfully due to occurring alcohols (xylitol, sorbitol, and arabinitol) [63] and fatty acids (linoleic, oleic, and erucic acids) (Table 3) [64]. Ergosterol, which is a secondary metabolite of *Aspergillus spp*., might come from its fungal cell membrane [65,66]. Furthermore, squalene and β-amyrin detected by GC×GC-TOFMS could be in the pathway of ergosterol synthesis [67].

Furthermore, 2,6-diisopropylnaphthalene detected by GC×GC-TOFMS (Table 3) could be caused by the usage of pesticides in pomegranate growth [68]. In a similar way, it is though that γ-tocopherol, which is an antioxidant, may be caused by the composition of pomegranate [69].

Due to the detection of 1,8-dihydroxynaphthalene by GC×GC-TOFMS (Table 3), our findings suggest that *A. carbonarius* might produce melanin from the polymerization of 1,8- dihydroxynaphthalene (DHN) from PP through the DHN pathway [30]. Furthermore, phthalic acid, mequinol, and galangin are known to be potent inhibitors of the DHN pathway [70,71,72]. Therefore, it is concluded that inhibitors could not surpass the melanin production. A potential metabolic pathway of melanin production by *Aspergillus carbonarius* from pomegranate pulp is illustrated in Figure S3.

To date, several species of *Aspergillus* such as *A. niger*, *A. flavus*, *A. tamarii*, *A. terreus*, *A. tubingensis*, *A. sydowii* and *A. fumigatus* were investigated for melanin production [30,31,60]. However, there is very little information about melanin production by *A. carbonarius*. Babitskaya et al. [55] studied melanin production by *A. carbonarius* on Czapek’s medium and they determined that melanin belongs to the DHN pathway. Another study on antioxidant properties of melanin produced by *A. carbonarius* was conducted by Shcherba et al. [73]. In accordance with these studies, it was thought that *A. carbonarius* could produce melanin from pomegranate pulp, which is an economical way, and that the produced melanin contains antioxidant. When evaluated together with SEM results, it was determined that the localization of this melanin was conidia.

Fungal melanin has attracted interest due to its potential usage in many industries including nanotechnology, biomedicine, dermo-cosmetics, and materials science [56]. However, *Aspergillus carbonarius* is also known as a producer of ochratoxin A (OTA) [74]. For this reason, further research on melanin production, OTA level and physical OTA removal methods such as filtration and pressure needs to be undertaken. It is known that OTA biosynthesis in *A. carbonarius* is driven by polyketide synthase (pks) and nonribosomal peptide synthetase genes (NRPS) [75]. The inactivation of these genes could be another way to eliminate the ability of this fungus to produce OTA [76] for a techno-economic way of producing pigment from pomegranate pulp.

In addition, GC×GC-TOFMS results show that the production of fatty acids such as linoleic, erucic and oleic acids by *A. carbonarius* could be another option for by-product valorization for the food processing industry [7].

### 3.7. Potential Usage of Fungal Pigment in Textile Industry

Images of dyed and undyed fabric pieces without the mordanting process are shown in Figure S4A,B, respectively. The dyeing test result shows that pigment obtained from this study could serve as a source for the natural dyeing of wool textiles. Dyed fabric was washed in the washing machine at a temperature of 60 °C five times and it was observed that the color did not change (Figure S4B). Dyed and undyed fabric pieces with mordant processing are shown in Figure S4C,D, respectively. Color fastness to washing was good to excellent (grade 3–4). On the one hand, the observation hue for the pieces was yellowish brown and yellowish pink for without mordanting and with mordanting process, respectively. However, it is known that the hue of dyes can be changed by using mordants such as iron sulfate [77]. Furthermore, colorimetric parameters such as dyeing rate constant, half-time of dyeing, and values of pigment uptake are worth further investigation.

On the other hand, the thermal stability of pigment can affect the coloring. However, it is known that thermal degradation of melanin occurs at very high temperatures (above 150 °C) [78]. Therefore, it is thought that the temperatures throughout our experimental studies (60 and 93 °C) after fermentation could not affect the melanin itself.

## 4. Conclusions

The results of our work reveal that pomegranate pulps derived from the food processing industry have a high potential for pigment production by *A. carbonarius*. The higher production of yellow pigment (61.84 ± 2.16 AU/g dfm) was obtained for 15 d at initial substrate pH of 6.5 under solid-state fermentation conditions. Moreover, our findings suggest that *A. carbonarius* might produce melanin due to the detection of 1,8-dihydroxynaphthalene by GC×GC-TOFMS and this will be investigated in the future by LC-MS-ToF. Optimal conditions such as incubation time, fermentation strategy, initial pH, and particle size of substrate findings in the current study can give basic information for the scaled-up production of bio-pigment production by filamentous fungus *Aspergillus carbonarius.* However, further studies are needed in order to detect potential utilization industrial areas of this bio-pigment.

## Figures and Tables

**Table 1:** Elemental analysis of unfermented pulps.

Type of Pulp	C (%)	H (%)	N (%)	S (%)	C:N
Black carrot	29.79	4.27	1.32	0.00	22.57/1
Red beet	37.54	5.43	1.82	0.00	20.63/1
Pomegranate	50.94	6.08	3.02	0.02	16.87/1
Apple	52.12	7.64	4.52	0.11	11.53/1

C: Carbon, H: Hydrogen, N: Nitrogen, S: Sulphur, C:N: carbon to nitrogen ratio.

**Table 2:** Elemental analysis of unfermented and fermented pomegranate pulp

Sample	C (%)	H (%)	N (%)	S (%)	C:N
Unfermented PP	50.94	6.08	3.02	0.02	16.87/1
Fermented PP	42.46	5.09	1.60	0.00	26.54/1

C: Carbon, H: Hydrogen, N: Nitrogen, S: Sulphur, C:N: carbon to nitrogen ratio.

**Table 3:** Chemical compounds determined by GC×GC-TOFMS

Name of Compounds	Chemical Structure	Formula	Classification	S
Xylitol	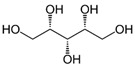	C_5_H_12_O_5_	Alcohol	951
L-Arabinitol	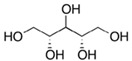	C_5_H_12_O_5_	Alcohol	946
Sorbitol	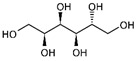	C_6_H_14_O_6_	Alcohol	796
Ethanol	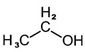	C_2_H_5_OH	Alcohol	949
Linoleic acid	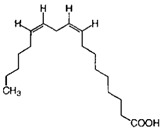	C_18_H_32_O_2_	Fatty acid	851
Oleic acid	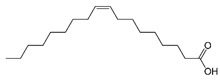	C_18_H_34_O_2_	Fatty acid	829
Erucic acid	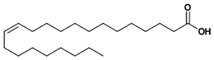	C_22_H_42_O_2_	Fatty acid	753
Ergosterol	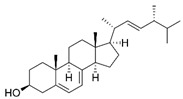	C_28_H_44_O	Sterol	794
Squalene	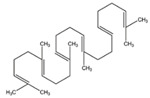	C_30_H_50_	Triterpene	778
β-Amyrin	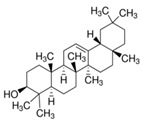	C_30_H_50_O	Triterpene	887
2,6-diisopropylnaphthalene	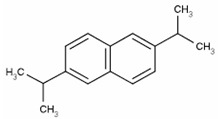	C_16_H_26_	Naphthalene	796
γ-Tocopherol	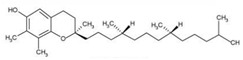	C_28_H_48_O_2_	Vitamin E	899
Galangin	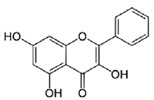	C_15_H_10_O_5_	Flavonoid	754
Phthalic acid	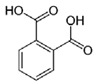	C_6_H_4_(CO_2_)	Dicarboxylic acid	884
Mequinol (4-methoxyphenol)	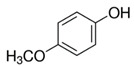	C_7_H_8_O_2_	Methoxyphenol	723
1,8-dihydroxynaphthalene	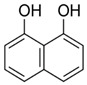	C_10_H_8_O_2_	Naphthalene	941

S: similarity.

## Supplementary Materials

The following are available online at https://www.mdpi.com/2309-608X/6/4/240/s1, Figure S1: Dried (A) apple pulp, (B) black carrot pulp, (C) pomegranate pulp, (D) red beet pulp, Figure S2: Flow chart of experiments (A) Selection of fungal species and pulp type, (B) Effect of fermentation strategy and incubation period, (C) Effect of substrate particle size, (D) Effect of initial substrate pH, (E) Pigment production at optimal conditions, Figure S3: Potential metabolic pathway of melanin production by *Aspergillus carbonarius* from pomegranate pulp, Figure S4: Images of (A) dyed and (B) undyed fabric pieces without mordanting, (C) dyed fabric pieces with mordanting, (D) undyed fabric pieces with mordanting (Dyeing of cotton fabric made with a fungal pigmented extract obtained by cultivation of *Aspergillus carbonarius* during 15 days on pomegranate pulp).

## References

[1] Vendruscolo F., Luise Müller B., Esteves Moritz D., de Oliveira D., Schmidell W., Luiz Ninow J. (2013). Thermal stability of natural pigments produced by *Monascus ruber* in submerged fermentation. Biocatal. Agric. Biotechnol..

[2] Shahid M., ul-Islam S., Mohammad F. (2013). Recent advancements in natural dye applications: A review. J. Clean. Prod..

[3] Nigam P.S., Luke J.S. (2016). Food additives: Production of microbial pigments and their antioxidant properties. Curr. Opin. Food Sci..

[4] Karger-Kocsis J. (1994). Paints, coatings and solvents. Compos. Sci. Technol..

[5] Barnett J.R., Miller S., Pearce E. (2006). Colour and art: A brief history of pigments. Opt. Laser Technol..

[6] Downham A., Collins P. (2000). Colouring our foods in the last and next millennium. Int. J. Food Sci. Technol..

[7] Panesar R., Kaur S., Panesar P.S. (2015). Production of microbial pigments utilizing agro-industrial waste: A review. Curr. Opin. Food Sci..

[8] US FDA (2016). Laws & Regulations-FDA Authority over Cosmetics: How Cosmetics Are not FDA-Approved, but Are FDA-Regulated. https://www.fda.gov/cosmetics/cosmetics-laws-regulations/fda-authority-over-cosmetics-how-cosmetics-are-not-fda-approved-are-fda-regulated.

[9] FAO. WHO (2017). Safety Evaluation of Certain Food Additives. https://apps.who.int/iris/bitstream/handle/10665/258934/9789241660730-eng.pdf;jsessionid=CEC17CE6D1D291C848E8289CFCD2A713?sequence=1.

[10] Embaby A.M., Hussein M.N., Hussein A. (2018). Monascus orange and red pigments production by *Monascus purpureus* ATCC16436 through co-solid state fermentation of corn cob and glycerol: An eco-friendly environmental low cost approach. PLoS ONE.

[11] Gupta N., Poddar K., Sarkar D., Kumari N., Padhan B., Sarkar A. (2019). Fruit waste management by pigment production and utilization of residual as bioadsorbent. J. Environ. Manag..

[12] Liu J., Luo Y., Guo T., Tang C., Chai X., Zhao W., Bai J., Lin Q. (2020). Cost-effective pigment production by *Monascus purpureus* using rice straw hydrolysate as substrate in submerged fermentation. J. Biosci. Bioeng..

[13] Thejus P.K., Krishnapriya K.V., Nishanth K.G. (2021). A cost-effective intense blue colour inorganic pigment for multifunctional cool roof and anticorrosive coatings. Sol. Energy Mater. Sol. Cells.

[14] Aruldass C.A., Dufossé L., Ahmad W.A. (2018). Current perspective of yellowish-orange pigments from microorganisms—A review. J. Clean. Prod..

[15] Tirumale S., Wani N.A., Gehlot P., Singh J. (2018). Biopigments: Fungal Pigments. Fungi and Their Role in Sustainable Development: Current Perspectives.

[16] Narsing Rao M.P., Xiao M., Li W.-J. (2017). Fungal and bacterial pigments: Secondary metabolites with wide applications. Front. Microbiol..

[17] Sen T., Barrow C.J., Deshmukh S.K. (2019). Microbial pigments in the food industry-Challenges and the way forward. Front. Nutr..

[18] Venil C.K., Zakaria Z.A., Ahmad W.A. (2013). Bacterial pigments and their applications. Process Biochem..

[19] Nirlane da Costa Souza P., Luiza Bim Grigoletto T., Alberto Beraldo de Moraes L., Abreu L.M., Henrique Souza Guimarães L., Santos C., Ribeiro Galvão L., Gomes Cardoso P. (2016). Production and chemical characterization of pigments in filamentous fungi. Microbiology.

[20] Lopes F.C., Tichota D.M., Pereira J.Q., Segalin J., de Oliveira Rios A., Brandelli A. (2013). Pigment Production by Filamentous Fungi on Agro-Industrial Byproducts: An Eco-Friendly Alternative. Appl. Biochem. Biotechnol..

[21] Karimi S., Mahboobi Soofiani N., Mahboubi A., Taherzadeh M. (2018). Use of Organic Wastes and Industrial By-Products to Produce Filamentous Fungi with Potential as Aqua-Feed Ingredients. Sustainability.

[22] Dufossé L., Fouillaud M., Caro Y., Mapari S.A.S., Sutthiwong N. (2014). Filamentous fungi are large-scale producers of pigments and colorants for the food industry. Curr. Opin. Biotechnol..

[23] Caro Y., Venkatachalam M., Lebeau J., Fouillaud M., Dufossé L., Merillon J.M., Ramawat K. (2017). Pigments and Colorants from Filamentous Fungi. Fungal Metabolites.

[24] Takahashi J.A., Carvalho S.A., Méndez-Vilas A. (2010). Nutritional potential of biomass and metabolites from filamentous fungi. Current Research, Technology and Education Topics in Applied Microbiology and Microbial Biotechnology.

[25] Teixeira M.F.S., Martins M.S., da Silva J.C., Kirsch L.S., Fernandes O.C.C., Carneiro A.L.B., de Conti R., Durán N. (2012). Amazonian biodiversity: Pigments from *Aspergillus* and *Penicillium*-characterizations, antibacterial activities and their toxicities. Curr. Trends Biotechnol. Pharm..

[26] Mostafa M.E., Saad Abbady M. (2014). Secondary Metabolites and Bioactivity of the *Monascus* Pigments Review Article. Glob. J. Biotechnol. Biochem..

[27] Heo Y.M., Kim K., Kwon S.L., Na J., Lee H., Jang S., Kim C.H., Jung J., Kim J.-J. (2018). Investigation of Filamentous Fungi Producing Safe, Functional Water-Soluble Pigments. Mycobiology.

[28] Saravanan A., Jayasree R., Senthil Kumar P., Varjani S., Hemavathy R.V., Jeevanantham S., Yaashikaa P.R. (2020). Production of pigment using *Aspergillus tamarii*: New potentials for synthesizing natural metabolites. Environ. Technol. Innov..

[29] Taniwaki M.H., Pitt J.I., Magan N. (2018). *Aspergillus* species and mycotoxins: Occurrence and importance in major food commodities. Curr. Opin. Food Sci..

[30] Pal A.K., Gajjar D.U., Vasavada A.R. (2014). DOPA and DHN pathway orchestrate melanin synthesis in *Aspergillus* species. Med. Mycol..

[31] Geib E., Gressler M., Viediernikova I., Hillmann F., Jacobsen I.D., Nietzsche S., Hertweck C., Brock M. (2016). A non-canonical melanin biosynthesis pathway protects *Aspergillus terreus* conidia from environmental stress. Cell Chem. Biol..

[32] Narendrababu B.N., Shishupala S. (2017). Spectrophotometric detection of Pigments from Aspergillus and Penicillium isolates. J. Appl. Biol. Biotechnol..

[33] Bouras H.D., Yeddou A.R., Bouras N., Hellel D., Holtz M.D., Sabaou N., Chergui A., Nadjemi B. (2017). Biosorption of Congo red dye by *Aspergillus carbonarius* M333 and *Penicillium glabrum* Pg1: Kinetics, equilibrium and thermodynamic studies. J. Taiwan Inst. Chem. Eng..

[34] Kantifedaki A., Kachrimanidou V., Mallouchos A., Papanikolaou S., Koutinas A.A. (2018). Orange processing waste valorisation for the production of bio-based pigments using the fungal speciess *Monascus purpureus* and *Penicillium purpurogenum*. J. Clean. Prod..

[35] Agboyibor C., Kong W.-B., Chen D., Zhang A.-M., Niu S.-Q. (2018). *Monascus* pigments production, composition, bioactivity and its application: A review. Biocatal. Agric. Biotechnol..

[36] Babitha S., Soccol C.R., Pandey A. (2007). Solid-state fermentation for the production of *Monascus* pigments from jackfruit seed. Bioresour. Technol..

[37] Johns M.R., Stuart D.M. (1991). Production of pigments by *Monascus purpureus* in solid culture. J. Ind. Microbiol..

[38] De Carvalho J.C., Cardoso L.C., Ghiggi V., Woiciechowski A.L., de Souza Vandenberghe L.P., Soccol C.R., Brar S.K., Dhillon G.S., Soccol C.R. (2014). Microbial Pigments. Biotransformation of Waste Biomass into High Value Biochemicals.

[39] Khan M.I., Ahmad A., Khan S.A., Yusuf M., Shahid M., Manzoor N., Mohammad F. (2011). Assessment of antimicrobial activity of Catechu and its dyed substrate. J. Clean. Prod..

[40] Shibila S.D., Nanthini A.U.R. (2019). Extraction and characterization of red pigment from *Talaromyces australis* and its application in dyeing cotton yarn. Int. Arch. App. Sci. Technol..

[41] Sankaran S., Khanal S.K., Jasti N., Jin B., Pometto A.L., Van Leeuwen J.H. (2010). Use of Filamentous Fungi for Wastewater Treatment and Production of High Value Fungal Byproducts: A Review. Crit. Rev. Environ. Sci. Technol..

[42] Akilandeswari P., Pradeep B.V. (2017). *Aspergillus terreus* Kmbf1501 A Potential Pigment Producer Under Submerged Fermentation. Int. J. Pharm. Pharm. Sci..

[43] Raman N.M., Shah P.H., Mohan M., Ramasamy S. (2015). Improved production of melanin from *Aspergillus fumigatus* AFGRD105 by optimization of media factors. AMB Express.

[44] Palacio-Barrera A.M., Areiza D., Zapata P., Atehortúa L., Correa C., Peñuela-Vásquez M. (2019). Induction of pigment production through media composition, abiotic and biotic factors in two filamentous fungi. Biotechnol. Rep..

[45] Said F.M., Brooks J., Chisti Y. (2014). Optimal C:N ratio for the production of red pigments by *Monascus ruber*. World J. Microbiol. Biotechnol..

[46] Dufossé L. (2016). Pigments, Microbial. Reference Module in Life Sciences. https://hal.archives-ouvertes.fr/hal-01734750/document.

[47] Caro Y., Anamale L., Fouillaud M., Laurent P., Petit T., Dufosse L. (2012). Natural hydroxyanthraquinoid pigments as potent food grade colorants: An overview. Nat. Prod. Bioprospect..

[48] Gmoser R., Ferreira J.A., Taherzadeh M.J., Lennartsson P.R. (2019). Post-treatment of Fungal Biomass to Enhance Pigment Production. Appl. Biochem. Biotechnol..

[49] Padmavathi T., Prabhudessai T. (2013). A Solid Liquid State Culture Method to Stimulate *Monascus* Pigments by Intervention of Different Substrates. Int. Res. J. Biol. Sci..

[50] Satyanarayana T., Deshmukh S.K., Johri B.N. (2017). Developments in Fungal Biology and Applied Mycology.

[51] Calvo A.M., Wilson R.A., Bok J.W., Keller N.P. (2002). Relationship between Secondary Metabolism and Fungal Development. Microbiol. Mol. Biol. Rev..

[52] Baker S.E., Bennett J., Machida M., Gomi K. (2007). An Overview of the Genus *Aspergillus*. The Aspergilli: Genomics, Medical Aspects, Biotechnology, and Research Methods.

[53] Teertstra W.R., Tegelaar M., Dijksterhuis J., Golovina E.A., Ohm R.A., Wösten H.A.B. (2017). Maturation of conidia on conidiophores of *Aspergillus niger*. Fungal Genet. Biol..

[54] Zalokar M. (1954). Studies on biosynthesis of carotenoids in *Neurospora crassa*. Arch. Biochem. Biophys..

[55] Babitskaya V.G., Shcherba V.V., Filimonova T.V., Grigorchyuk E.A. (2000). Melanin pigments from the fungi *Paecilomyces variotii* and *Aspergillus carbonarius*. Appl. Biochem. Microbiol..

[56] Pombeiro-Sponchiado S.R., Sousa G.S., Andrade J.C.R., Lisboa H.F., Gonçalves R.C.R., Blumenber M. (2017). Production of Melanin Pigment by Fungi and Its Biotechnological Applications. Melanin.

[57] Afshari M., Shahidi F., Mortazavi S.A., Tabatabai F., Es’haghi Z. (2015). Investigating the influence of pH, temperature and agitation speed on yellow pigment production by *Penicillium aculeatum* ATCC 10409. Nat. Prod. Res..

[58] Arora D.S., Chandra P. (2011). Antioxidant Activity of *Aspergillus fumigatus*. ISRN Pharmacol..

[59] Cho Y.J., Park J.P., Hwang H.J., Kim S.W., Choi J.W., Yun J.W. (2002). Production of red pigment by submerged culture of *Paecilomyces sinclairii*. Lett. Appl. Microbiol..

[60] Pihet M., Vandeputte P., Tronchin G., Renier G., Saulnier P., Georgeault S., Mallet R., Chabasse D., Symoens F., Bouchara J.-P. (2009). Melanin is an essential component for the integrity of the cell wall of *Aspergillus fumigatus* conidia. BMC Microbiol..

[61] Hernández F., Ibáñez M., Portolés T., Cervera M.I., Sancho J.V., López F.J. (2015). Advancing towards universal screening for organic pollutants in waters. J. Hazard. Mater..

[62] Hasnaoui N., Wathelet B., Jiménez-Araujo A. (2014). Valorization of pomegranate peel from 12 cultivars: Dietary fibre composition, antioxidant capacity and functional properties. Food Chem..

[63] Schiweck H., Bär A., Vogel R., Schwarz E., Kunz M., Dusautois C., Clement A., Lefranc C., Lüssem B., Moser M. (2012). Sugar Alcohols. Ullmann’s Encyclopedia of Industrial Chemistry.

[64] Sinha M., Weyda I., Sørensen A., Bruno K.S., Ahring B.K. (2017). Alkane biosynthesis by *Aspergillus carbonarius* ITEM 5010 through heterologous expression of Synechococcus elongatus acyl-ACP/CoA reductase and aldehyde deformylating oxygenase genes. AMB Express.

[65] Alcazar-Fuoli L., Mellado E., Garcia-Effron G., Lopez J.F., Grimalt J.O., Cuenca-Estrella J.M., Rodriguez-Tudela J.L. (2008). Ergosterol biosynthesis pathway in *Aspergillus fumigatus*. Steroids.

[66] Vadlapudi V., Borah N., Yellusani K.R., Gade S., Reddy P., Rajamanikyam M., Vempati L.N.S., Gubbala S.P., Chopra P., Upadhyayula S.M. (2017). Aspergillus Secondary Metabolite Database, a resource to understand the Secondary metabolome of *Aspergillus* genus. Sci. Rep..

[67] Gealt M.A. (1983). Isolation of p-Amyrin from the Fungus *Aspergillus nidulans*. J. Gen. Microbiol..

[68] Mohamed E.M. (2016). Flavoring and medicinal values of the yellow pigment produced by *Monascus ruber* 4066 species cultivated on static malt agar medium. Int. Res. J. Biochem. Biotechnol..

[69] Caligiani A., Bonzanini F., Palla G., Cirlini M., Bruni R. (2010). Characterization of a Potential Nutraceutical Ingredient: Pomegranate (*Punica granatum L.*) Seed Oil Unsaponifiable Fraction. Plant Foods Hum. Nutr..

[70] Yin S.J., Si Y.X., Qian G.Y. (2011). Inhibitory Effect of Phthalic Acid on Tyrosinase: The Mixed-Type Inhibition and Docking Simulations. Enzym. Res..

[71] Bandyopadhyay D. (2009). Topical treatment of melasma. Indian J. Dermatol..

[72] Lu Y.H., Tao L., Wang Z.T., Wei D.Z., Xiang H.B. (2007). Mechanism and inhibitory effect of galangin and its flavonoid mixture from *Alpinia officinarum* on mushroom tyrosinase and B16 murine melanoma cells. J. Enzym. Inhib. Med. Chem..

[73] Shcherba V.V., Babitskaya V.G., Kurchenko V.P., Ikonnikova N.V., Kukulyanskaya T.A. (2000). Antioxidant Properties of Fungal Melanin Pigments. Appl. Biochem. Microbiol..

[74] Zeidan R., Ul-Hassan Z., Al-Thani R., Migheli Q., Jaoua S. (2019). In-Vitro Application of a *Qatari Burkholderia cepacia* species (QBC03) in the Biocontrol of Mycotoxigenic Fungi and in the Reduction of Ochratoxin A biosynthesis by Aspergillus carbonarius. Toxins.

[75] Gallo A., Bruno K.S., Solfrizzo M., Perrone G., Mulè G., Visconti A., Baker S.E. (2012). New Insight into the Ochratoxin A Biosynthetic Pathway through Deletion of a Nonribosomal Peptide Synthetase Gene in *Aspergillus carbonarius*. Appl. Environ. Microbiol..

[76] Castellá G., Bragulat M.R., Puig L., Sanseverino W., Cabañes F.J. (2018). Genomic diversity in ochratoxigenic and non ochratoxigenic strains of *Aspergillus carbonarius*. Sci. Rep..

[77] Moghaddam M.K., Adivi M.G., Dehkord M.T. (2019). Effect of Acids and Different Mordanting Procedures on Color Characteristics of Dyed Wool Fibers Using Eggplant Peel (*Solanum melongena L*). Prog. Color Colorants Coat..

[78] Pralea I.E., Moldovan R.C., Petrache A.M., Ilieș M., Hegheș S.C., Ielciu I., Nicoară R., Moldovan M., Ene M., Radu M. (2019). From Extraction to Advanced Analytical Methods: The Challenges of Melanin Analysis. Int. J. Mol. Sci..

