# Supplementary material for: Production of Bio-Based Pigments from Food Processing Industry By-Products (Apple, Pomegranate, Black Carrot, Red Beet Pulps) Using Aspergillus carbonarius
Source: J Fungi (Basel). 2020 Oct 22;6(4):240. doi: 10.3390/jof6040240 (PMC7712229; doi:10.3390/jof6040240)
Supplement: Supplementary file 1 [file jof-06-00240-s001.pdf]

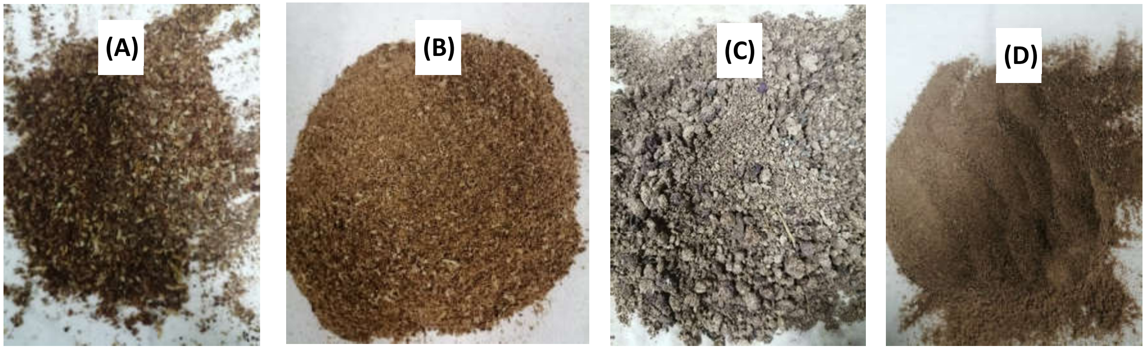

Figure S1. Dried **(A)** apple pulp, **(B)** black carrot pulp, **(C)** pomegranate pulp, **(D)** red beet pulp

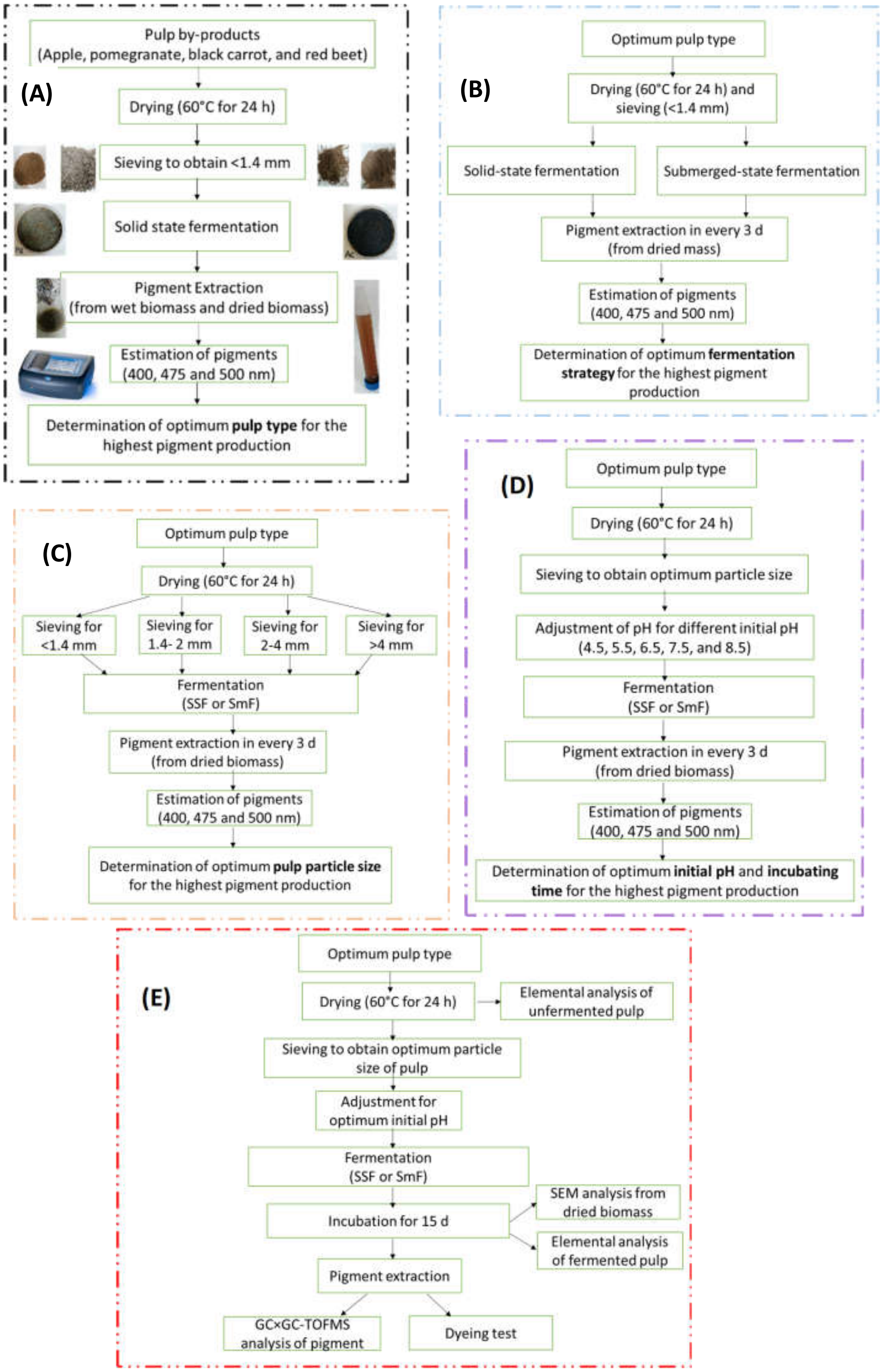

Figure S2. Flow chart of experiments **(A)** Selection of fungal species and pulp type, **(B)** Effect of fermentation strategy and incubation period, **(C)** Effect of substrate particle size, **(D)** Effect of initial substrate pH, **(E)** Pigment production at optimal conditions

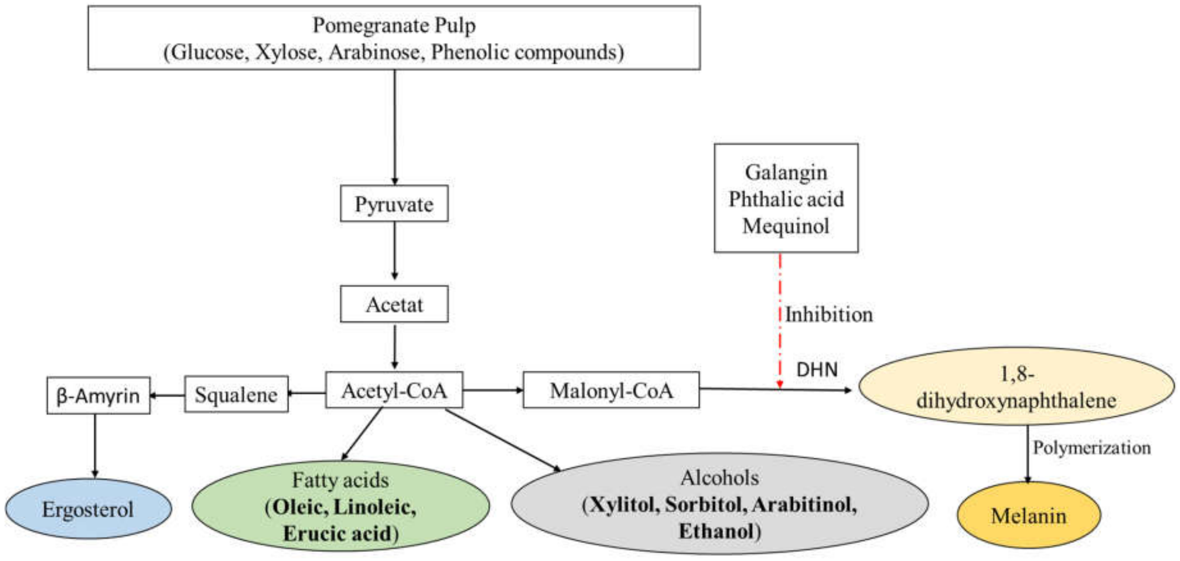

Figure S3. Potential metabolic pathway of melanin production by *Aspergillus carbonarius* from pomegranate pulp (adapted from Vadlapudi et al., 2017)

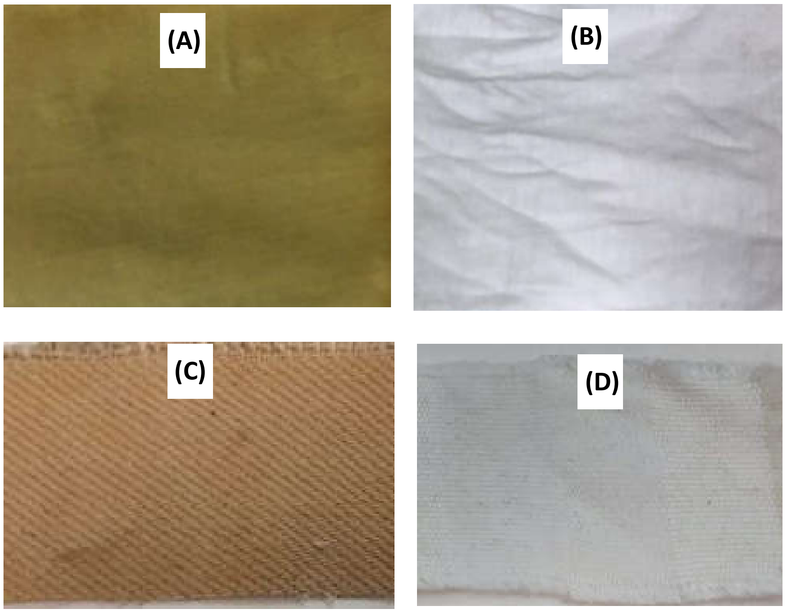

Figure S4. Images of **(A)** dyed and **(B)** undyed fabric pieces without mordanting, **(C)** dyed fabric pieces with mordanting, **(D)** undyed fabric pieces with mordanting (Dyeing of cotton fabric made with a fungal pigmented extract obtained by cultivation of *Aspergillus carbonarius* during 15 days on pomegranate pulp)
